# Long-term use of ceftriaxone sodium induced changes in gut microbiota and immune system

**DOI:** 10.1038/srep43035

**Published:** 2017-02-21

**Authors:** Yanjie Guo, Xuefei Yang, Yane Qi, Shu Wen, Yinhui Liu, Shaoying Tang, Rongsheng Huang, Li Tang

**Affiliations:** 1 Department of Microecology, School of Basic Medical Science, Dalian Medical University, Dalian, Liaoning, China; 2 The second clinical college, Dalian Medical University, Dalian, Liaoning, China

## Abstract

Antibiotic administration, while facilitating clearance of targeted infections, also perturbs commensal microbial communities. Previous studies have all focused on the effects of short term use of antibiotics. Here, we focus on the effects of long term use of antibiotic on gut microbiota and immunity. BALB/c mice received saline or different doses of ceftriaxone sodium (100, 200 and 400 mg/mL) via daily gavage for 150 days. Alterations of fecal microbiota, small intestine histopathology, body weight, spleen index, serum IgG, mucus SIgA, IFN-γ/IL-4 ratio, CD4/CD8 ratio and CD4^+^CD25^+^ cells were evaluated. Long term ceftriaxone sodium administration resulted in gut microbiota dysbiosis, intestine histological lesions, growth inhibition, spleen index reducing. The immune defense ability reduced as serum IgG and mucus SIgA decreased significantly. Not only the immune defense, long term ceftriaxone administration also affected immune regulation. The IFN-γ/IL-4 and CD4/CD8 ratios increased, the CD4^+^CD25^+^ cells reduced on days 30 and 60 after ceftriaxone administration. However, after 90 days of ceftriaxone administration, the IFN-γ/IL-4, CD4/CD8 ratios and CD4^+^CD25^+^ cells restored, which indicated a new balance of immune regulation had been formed. Overall, these findings contribute to our understanding of long term antibiotic administration influencing gut microbiota and immunity.

In modern societies, widespread antibiotic administration is probably a major factor contributing to rapid changes in the gut microbiota. Several lines of evidence confirmed that antibiotic administration can result in gut microbiota dysbiosis. Broad-spectrum antibiotics can influence the abundance of roughly one third of the bacterial taxa in the gut, causing rapid and significant drops in taxonomic richness, diversity and evenness^1^. Repeated use of broad-spectrum antibiotic in the process of treating infections may cause more serious microbiota dysbiosis. A study of repeated use of antibiotic demonstrated that the distal gut microbiota changed among subjects and between the two courses within subjects after two courses of ciprofloxacin administration within 10 months^2^. Along similar lines, mouse models also showed that treatment with ampicillin, clindamycin or vancomycin induced microbiota dysbiosis^3^,^4^,^5^.

The commensal gut microbiota has a profound effect on immune system. The gut microbiota contributes to the development and differentiation of the mammalian immune system^6^. In fact, both the immune response and immune homeostasis are affected by gut microbiota. Data from germ free animals demonstrated that the serum IgG level was significantly lower than that in conventional mice. The ‘switch’ from IgM to IgG synthesis was suppressed, the IgG response to sheep red cell and dinitrophenylated bovine serum albumin was lower than that in conventional mice, which is a reflection of immaturity or deficiency of T cells^7^. Most recently, Zeng *et al*. reported that selective gut symbiotic bacterium were able to disseminate systemically to induce IgG response, which conferred protection against systemic infections^8^. Besides IgG, SIgA production attenuated markedly in germ-free mice, which can be restored to near normal levels over the course of several weeks after conventionalization with bacterial species that can establish stable residence in the intestine^9^. The gut microbiota could also operate as an important regulator of Th1/Th2 balance. The im-balanced Th1/Th2 cell ratio in germ free mice can be restored through colonization of *Bacteriodes fragilis*. Moreover, studies have shown that homeostatic T cell proliferation itself is driven by the microbiota or their penetrant molecules^10^. Colonic T regulatory (Treg) cell populations are shaped by intestinal-derived antigens^11^.

Depletion of commensal microbes and changing the microbiota composition by antibiotic administration affects the immune system. Treatment with amoxicillin for 3 weeks decreased lactic acid bacteria but increased fecal *enterobacteria* counts in pigs’ jejunum. Amoxicillin-induced microbiota changes reduced the mean total serum IgM concentration^12^. Depletion or deletion of bacterial communities with a cocktail of antibiotics containing ampicillin, gentamicin, neomycin, metronidazole and vancomycin was associated with increased interleukin-4 (IL-4) circulating levels, exaggerated type 2 T helper cell (Th2 cell) responses and elevated serum immune globulin E (IgE) levels^13^. Our previous study showed that short term (for 7 days) use of ceftriaxone-induced microbiota dysbiosis resulted in decreased SIgA secretion and increased inflammatory cytokines levels^14^.

It is clear that antibiotics-induced microbiota dysbiosis disrupts the immune system. However, till now influences of antibiotics on bacterium and host immune system are all short-term administrations, the influences of long-term use of antibiotics are unclear. Due to medical treatment (repeated use of antibiotics) and utilization in farm animals and crops, the human microbiome is always continuously exposed to antibiotics. Therefore, it is very interesting to explore the influences of long-term administration of antibiotics on bacterium and immune system. In this study, BALB/c mice were treated with ceftriaxone for a long period (5 months), feces microbiota, mucus SIgA, circulating IgG, IFN-γ/IL-4 ratio, CD_4_^+^/CD_8_^+^ ratio, and CD4^+^CD25^+^ cells were examined to illustrate the changes of gut microbiota and immune function. This study helps our understanding of the influences of long-term antibiotic exposure on host gut microbiota and immune system.

## Results

### Dynamic changes of intestinal microbiota in ceftriaxone-treated mice based on DGGE fingerprint

**Figure 1:**
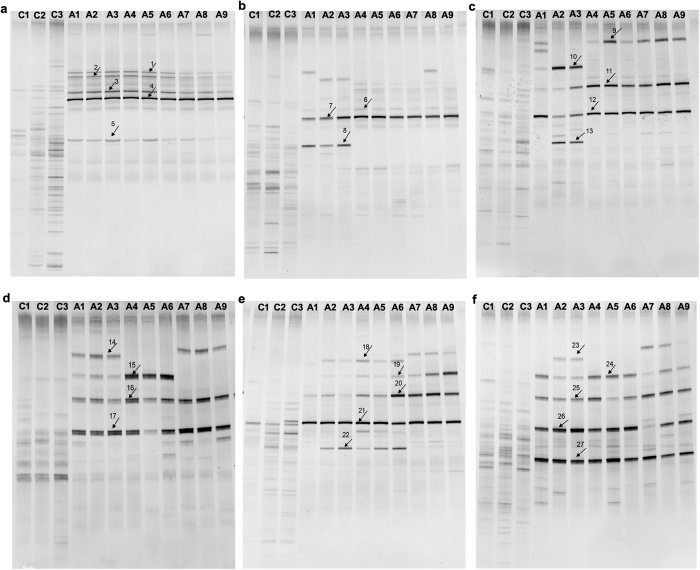
16S rRNA gene V3 region PCR-DGGE profiles in the fecal microbiota of control group and ceftriaxone treated groups (control group: C1–C3; 100 mg/mL ceftriaxone group: A1–A3; 200 mg/mL ceftriaxone group: A4–A6; 400 mg/mL ceftriaxone group: A7–A9, each group n = 3). Different time points of fecal sample were examined ((**a**) on day 8; (**b**) on day 30; (**c**) on day 60; (**d**) on day 90; (**e**) on day 120; (**f**) on day 150). Arrows 1–27 were the bands selected for sequence.

PCR-DGGE fingerprint analysis for predominant bacteria was used to capture the structural response in gut microbiota after ceftriaxone treatment. The bacterial diversity reduced in the ceftriaxone treatment groups compared with the control group. Contrary to the decreasing pattern of overall bacterial population, several bands in the antibiotic-treated samples were more prominent (Fig. 1). These strong bands were selected and cut for sequencing. The results were shown in Table 1. The bands were assigned to bacterial species based on the highest sequence similarity match to GeneBank sequences obtained by BLAST analysis. On day 8, the strong bands in antibiotic-treated samples were identified as *Enterococcus* genus (bands 1–4) and *Clostridium* species (band 5). On day 30, the number of bands in ceftriaxone treatment groups was further reduced, leaving only 3 prominent bands. They were identified as *Enterococcus* genus and *Robinsoniella* genus (bands 6–8). On day 60, the prominent bands were identified as *Anaeroplasma, Enterococcus, Robinsoniella* and *Escherichia* genus (bands 9–13). On day 90, the prominent bands in ceftriaxone treatment groups were identified as *Anaeroplasma, Enterococcus* and *Robinsoniella* genus (bands 14–17). On days 120 and 150, the prominent bands in ceftriaxone treatment groups were identified as *Anaeroplasma, Enterococcus, Robinsoniella* and *Escherichia* genus (bands 18–22). On the whole, the *Enterococcus* genus was the dominate bacterium throughout the experiments. The *Robinsoniella* genus became dominate after 30 days of ceftriaxone treatment. The *Anaeroplasma* genus became dominate after 60 days of ceftriaxone treatment.

### Effects of ceftriaxone treatment on histopathology of the intestinal mucosa

**Figure 2:**
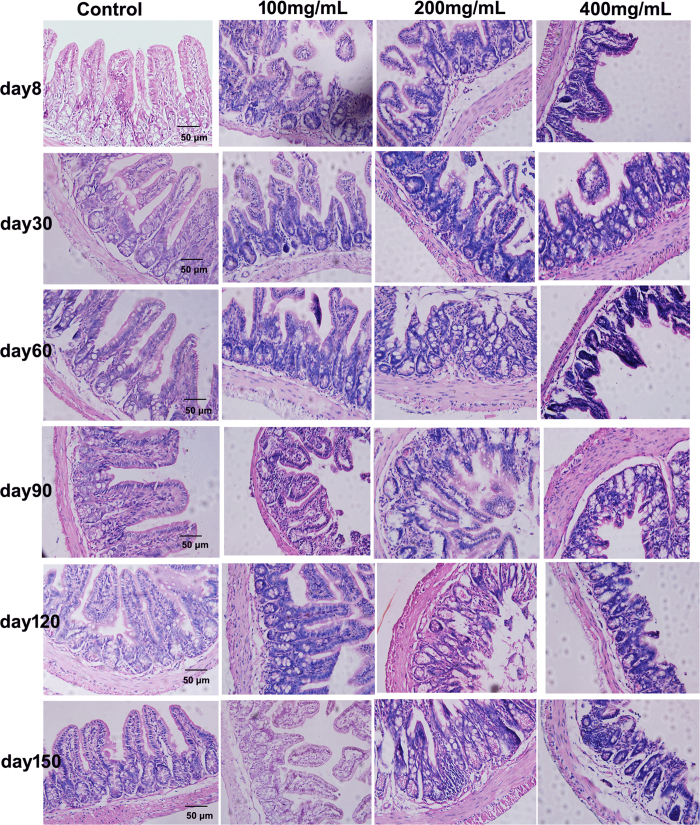
Light micrograph of the small intestine stained with hematoxylin and eosin for different time points. (**a**) control group, (**b**) 100 mg/mL ceftriaxone group, (**c**) 200 mg/mL ceftriaxone group, (**d**) 400 mg/mL ceftriaxone group. Magnification (400×), scale bar, 50 μm.

Histological cross-sections of distal small intestine from all mice are shown in Fig. 2. Characteristic features in control mice included well-shaped, elongated villi lined with columnar epithelia having regularly-spaced dark-staining nuclei and evenly distributed dark-staining nuclei in lamina propia. Cross-sections of ceftriaxone treated mice revealed loss of regular villi structure, lack of regularly distributed columnar epithelial cell nuclei lining villi, shortened and irregular villi, and concentration of dark-staining nuclei near the villi base. The villi lesion in 400 mg/mL group was more serious than 200 and 100 mg/mL groups (The average length of villi: 100 mg/mL 121 ± 8.9 μm, 200 mg/mL 106 ± 5.5 μm, 400 mg/mL 82 ± 8.3 μm, P = 0.01).

### Effects on body weight and spleen index of mice after long-term ceftriaxone treatment

**Figure 3:**
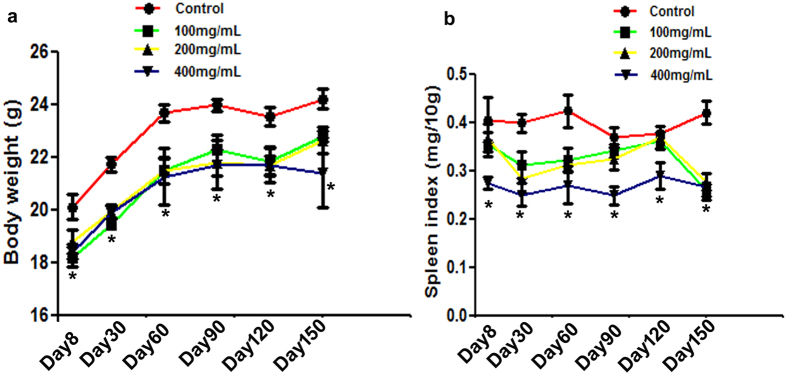
Effects of long term ceftriaxone administration on body weight and spleen index. (**a**) mice body weight at different time points, (**b**) mice spleen index at different time points (each group n = 6), *p < 0.05 compared to all other groups.

To examine the effect of ceftriaxone treatment on body weight, all mice body weight was weighed at different time points. The results showed that after 150 days of breeding, all mice body weight was increased (Fig. 3a). However, the average body weight of ceftriaxone-treated mice was significantly lower than that of control mice (body weight: control 24.2 ± 0.36 g, 100 mg/mL 22.8 ± 0.2 g, 200 mg/mL 22.6 ± 0.5 g, 400 mg/mL 21.4 ± 1.3 g, P = 0.011). There were no significant changes of body weight among different dosages of ceftriaxone treated groups after 150 days of ceftriaxone treatments (P = 0.414).

To evaluate the changes of immune organ after long term ceftriaxone treatment, the spleen index was calculated. The results indicated that the spleen index fluctuated within a certain range during the experiment (Fig. 3b). The spleen index in control group, 100 mg/mL, 200 mg/mL and 400 mg/mL ceftriaxone treatment group fluctuated from 3.69 to 4.2, 2.74 to 3.63, 2.7 to 3.68 and 2.48 to 2.9, respectively. The spleen index was lower in 400 mg/mL ceftriaxone treatment group at all time points when compared with control mice (main effect of ceftriaxone dosage P < 0.001).

### The concentrations of immune globulin and cytokines after long term ceftriaxone treatment

**Figure 4:**
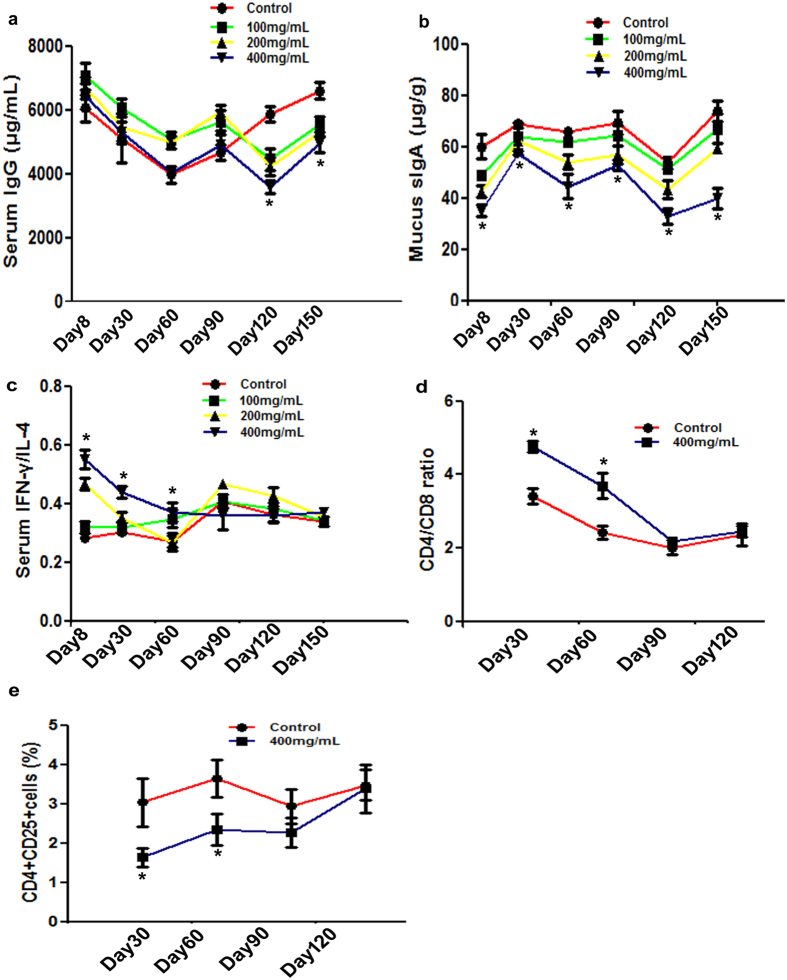
Effects of long term ceftriaxone administration on immunity. (**a**) Serum IgG concentration, (**b**) mucus SIgA concentration, (**c**) serum IFN-γ/IL-4 ratio, (**d**) serum CD4/CD8 ratio, (**e**) CD4^+^CD25^+^ cells in the blood, (each group n = 6), *p < 0.05 compared to all other groups. ^#^p < 0.05 when compared with control group.

The serum concentration in IgG was analyzed at all time points (Fig. 4a). The results showed that there were no significant variations among groups before day 90. However, significant decreasing of IgG concentrations were observed in a dose dependent manner in ceftriaxone treated groups on days 120 and 150 when compared with the control group (time × dosage interaction P = 0.0003).

As for intestinal mucus SIgA concentrations, the concentrations of intestinal mucus SIgA were significantly decreased in a dose dependent manner in ceftriaxone treated groups when compared with the control group (main effect of ceftriaxone dosage P < 0.0001) (Fig. 4b).

IFN-γ and IL-4 are considered to be the characteristic cytokines produced by Th1 and Th2 cells, respectively. IFN-γ/IL-4 cytokine ratio was used by researchers to estimate Th1/Th2 balance^15^,^16^. The IFN-γ/IL-4 ratio showed no statistically significant difference between 100 mg/mL ceftriaxone treatment group and control group throughout the experiment. In 200 mg/mL ceftriaxone treatment group, the IFN-γ/IL-4 ratio was significantly higher than that in control group on day 8 (P = 0.006). In 400 mg/mL ceftriaxone treatment group, the IFN-γ/IL-4 ratios were significantly higher than those in control group on days 8 and 30 (P = 0.0006 and 0.001, respectively); On day 60, the IFN-γ/IL-4 ratios was still higher than that in control group, but not significantly; On day 90, the IFN-γ/IL-4 ratio was slight lower than that in control group; On days 120 and 150, there were no significant statistical differences among groups (Fig. 4c).

### Effects of long term ceftriaxone administration on peripheral blood CD4/CD8 ratio and CD4^+^CD25^+^ cells

CD4/CD8 ratio is a sensitive indicator of clinical diagnosis to determine immune dysfunction. The increase of this ratio indicates cellular immune function in a “hyperactive” state. In this study, the CD4/CD8 ratio in peripheral blood was higher in 400 mg/mL ceftriaxone treatment group on days 30 and 60 when compared with the control group (P = 0.002 and 0.03, respectively). On days 90 and 120, there were no significant statistical differences of CD4/CD8 ratio between control group and 400 mg/mL ceftriaxone treatment group (Fig. 4d).

CD4^+^CD25^+^ cells are important immunological modulatory cells that display a crucial role in the immunological tolerance. The number of CD4^+^CD25^+^ cells were significant lower on days 30 and 60 in 400 mg/mL ceftriaxone treatment group when compared with the control group (P = 0.042 and 0.038, respectively). On days 90 and 120, there were no significant statistical difference of CD4^+^CD25^+^ cells number between control group and 400 mg/mL ceftriaxone treatment group (Fig. 4e).

## Discussion

The extent of antibiotic impact on the microbiota depends on the spectrum of antibiotic, the dose and duration of treatment, and the route of administration. In this study, BALB/c mice were administered a gavage of the broad-spectrum antibiotic ceftriaxone sodium for 150 days. The bacterial diversity alterations in the gut after using ceftriaxone were proved by PCR-DGGE. The fecal bacterial diversity reduced significantly in ceftriaxone treatment groups especially in 400 mg/mL group when compared with the control group throughout the experiment. The reduction in gut microbiota diversity is associated with disease as loss of microbiota diversity was observed in digestive diseases such as Crohn’s disease, irritable bowel syndrome, colorectal cancer and non-digestive diseases such as autism^17^,^18^,^19^,^20^. It has been proposed that this microbial functional diversity would have been beneficial for the host since it might have shaped the adaptive immune system^21^. From this point of view, in this study, the decreasing of gut microbiota diversity after ceftriaxone administration may be one of the reasons for the changes of immunity. Further studies are needed to illustrate the relationship between microbiota diversity and its consequences on the immune system.

In this study, ceftriaxone treatment reduced the microbial diversity but increased single bacterial species. On days 8 and 30, *Enterococcus* and *Robinsoniella* genus became dominant microflora in the gut. After 60 days of ceftriaxone treatment, *Anaeroplasma, Enterococcus, Robinsoniella* and *Escherichia* genus became dominant in the gut. During the long term treatment, *Anaeroplasma, Robinsoniella* and *Escherichia* genus gradually became ceftriaxone resistant, and became dominant genus. *Enterococcus* genus was the dominant microflora during the experiment because ceftriaxone could induce *Enterococcal* expansion in the mouse intestine^22^. *E. faecium* is a highly recombining, multi-resistant organism, which appears both to have initially adapted to the hospital environment and to have repeatedly evolved new clones that have competed with each other, while frequently gaining and losing vancomycin resistance^23^. *Enterococcus faecium* is a major cause of hospital-acquired infections, especially in severely immunocompromised patients^24^. The increased dominance of *E. faecium* within the gut microbiota subsequently results in enhanced cross-transmission via surfaces and inter-personal contacts and invasive disease in a subset of colonized patients^25^. Another bacterial species becomes dominate after ceftriaxone treatment in this study is *Robinsoniella peoriensis. Robinsoniella peoriensis* is a recently described anaerobic, spore-forming, gram positive bacillus originally recovered from swine-manure and clinical human samples. Antibiotic resistance was observed for cefotaxime, clindamycin and moxifloxacin^26^. In this study, the expansion of *Enterococcus* and *Robinsoniella* genus indicated that they were resistant to ceftriaxone and may be the reason for hospital-acquired infections. Clinicians should take into account the emergence of *Enterococcus* and *Robinsoniella* resistant strains in patients with repeated ceftriaxone sodium application.

Long-term antibiotic administration inducing growth inhibition and spleen index decreasing were observed in this study. Spleen index can directly reflect the level of immune functions in the body. Effects of drugs on the spleen index can be used as the preliminary indicator for the study on immunopharmaco-logical mechanisms in animals^27^. Here, the results showed that the spleen indexes were decreased markedly in the antibiotic groups in a dose dependent manner, which suggested that ceftriaxone can damage the spleen to a certain extent, thereby weakening the immune function.

In order to investigate the effects of long term antibiotic treatment on immunity, the concentration of innate immunity factor IgG in serum was explored. Before day 90, the concentrations of IgG did not vary significantly between control mice and antibiotic treated mice. Nevertheless, mean IgG concentrations were higher in antibiotic treated mice than those in control mice. The higher IgG concentrations could be a result of leaky guts, increased bacterial translocation, and at the same time might serve as a compensatory protective mechanism^8^. On days 120 and 150, serum IgG concentrations decreased significantly in antibiotic treated mice. Long-term gut microbiota dysbiosis might be an important mechanism to induce the decrease of serum IgG concentration, which has been supported by recent study stating that IgG was induced by symbiotic bacterium^8^. SIgA is the most abundant immunoglobulin found in intestinal mucus. Our results showed that SIgA secretion in ceftriaxone treated mice decreased significantly in a dose dependent manner when compared with control mice throughout the experiment, which suggested a significant reduction in the protective ability of the intestinal mucosa against exogenous bacterial infections.

Long term antibiotic administration affected both the immune defense and the immune regulation. From an evolutionary point of view, antibiotic application at the beginning posed a great blow to the immune system, which dramatically reduced the immunity. However, following long-term antibiotic administrating the body adjusted the immune system, improved the body’s immune function, which made the immune system obtain a new immune balance. In this study, the ratio of IFN-γ to IL-4, Th1, and Th2 cytokines, respectively, were higher in 400 mg/mL ceftriaxone treated mice than in the control mice before day 60. On day 90, the IFN-γ/IL-4 ratio was lower than that in control mice. On days 120 and 150, there were no significant statistical difference between groups. These findings indicated that the early stage of ceftriaxone administration probably shifted the Th1/Th2 balance toward Th1, then, triggered a short shift to Th2. Long term ceftriaxone administration resulting in a new balance of Th1 and Th2.

The CD4/CD8 ratio is considered a marker for both immune senescence and immune activation. The increase of this ratio indicated cellular immune function in a “hyperactive” state, such as organ transplant rejection, rheumatoid arthritis, and so on. In this study, the CD4/CD8 ratio increased on days 30 and 60 then decreased to normal on days 90 and 120 in 400 mg/mL ceftriaxone treatment group. The inflammation state after ceftriaxone administration may be one of the reasons to explain the increase of CD4/CD8 ratio, as circulating proinflammatory cytokines were elevated after ceftriaxone administration^14^. Inflammation has been gradually considered as a mechanism of immune defense and repair. Long term ceftriaxone administration induced a new balance of the immunity as the CD4/CD8 ratio restoration was observed in mice after 90 days of ceftriaxone administration.

Regulatory T lymphocytes (Tregs) can regulate or suppress the function of other immune cells. CD4^+^CD25^+^ cells, a type of Tregs, have been identified in mice as a distinct population of CD4^+^ T cells that constitutively express the interleukin (IL)-2 receptor α -chain (CD25)^28^,^29^. In this study, during the long term of ceftriaxone administration the CD4^+^CD25^+^ cells reduced significantly before day 60, then from day 90 to day 120, the CD4^+^CD25^+^ cells restored though still lower than those in control group, but the differences were not significant. These data suggested that long term ceftriaxone administration broke the immune balance at the beginning, however after adjustment, the immunity balance restored.

We do not know the exact mechanism underlying this change of immunity, but the antibiotic induced gut microbiota changes may be one of the reasons. Experiments in mice have revealed oral treatment with enrofloxacin early in life promotes Th2-mediated immune response^30^. Microbiota intervention through probiotic (*Lactobacillus casei variety rhamnosus*) restored the Th1/Th2 balance in trimellitic anhydride-induced atopic dermatitis mice^31^, implying microbiota changes impact the immunity from another angle. Moreover, different specific bacteria and bacterial products are capable of Treg cell induction. *Clostridia* and *Bacteroides fragilis* could be the most powerful inducers of Treg cells^32^,^33^. In this study, the gut microbiota diversity decreasing and the host immune system adjust to the long term symbiotic bacterium decreasing may be one of the reasons for the changes of immunity.

In conclusion, our data suggested that ceftriaxone administration induced histological lesions of small intestine, growth inhibition and decrease of spleen index. Long term ceftriaxone administration induced a reduction in commensal bacterium and overgrowth of antibiotic resistant bacterium, which had a great impact on both local immune response and systemic immune response. The concentrations of circulating IgG and intestinal mucus SIgA were decreased after long term ceftriaxone administration. Except for immune response, long term ceftriaxone administration also affected immune regulation. At the beginning of ceftriaxone administration (before day 60), the Th1/Th2 balance shifted toward Th1, CD4/CD8 ratio increased, CD4^+^CD25 ^+^ cells reduced. However, after day 90, the Th1/Th2 balance, CD4/CD8 ratio and CD4^+^CD25^+^ cells restored. These data indicated that long term ceftriaxone administration destroyed the original immune homeostasis and formed a new immune balance. Further studies are needed to investigate the influences of long term ceftriaxone administration on immune system.

## Materials and Methods

### Animals

Specific-pathogen free (SPF) level inbred female BALB/c mice (aged 6–8 weeks, weighing 18 ± 22 g) were provided by the Experimental Animal House of Dalian Medical University. The study protocol was approved by the Animal Care Committee of Dalian Medical University, China (SCXK-2013-0006) and conformed to the Guide for the Care and Use of Laboratory Animals. All animals were feed with commercial diet and tap water *ad libitum*. In this experiment, 144 female BALB/c rats were randomly assigned into 4 groups of 36 mice each, including a control group that received a gavage of normal saline, and three antibiotic groups that treated with 0.2 mL different doses of ceftriaxone sodium (100, 200 and 400 mg/mL) intragastrically twice a day with an interval of 6 h. For each time points (on days 8, 30, 60, 90, 120 and 150), 6 mice each group were sacrificed by cervical dislocation after inhalational anesthesia.

### DGGE analysis and sequence analysis

The DGGE analysis was performed according to descriptions of Joossens *et al*.^34^. To identify specific bands, bands were excised from the gel and sequenced (Takara, Japan). The sequences were compared directly with those in GeneBank by Blast search (NCBI, http://www.ncbi.nlm.nih.gov/).

### Morphological examination and histological analysis

The distal small intestine tissue sample was cut into ultra-thin cross sections, fixed in 10% neutral buffered formalin, embedded in paraffin and sectioned. Every 10th section (n = 6) was mounted on a glass slide, stained with hematoxylin/eosinand and analyzed using an Olympus DP73 microscope (Olympus, Tokyo, Japan) by two persons blinded to the origin of sections.

### Collection of biological samples and detection of SIgA, IgG, IFN-γ and IL-4

After mice were sacrificed, the small intestine was removed from mice. Intestinal mucus sample for SIgA analysis was collected according to our previous work [10]. Blood samples were obtained and sera were kept at −80 °C for subsequent experiments. The concentrations of SIgA, IgG, IFN-γ and IL-4 were determined using enzyme-linked immunosorbent assay (ELISA) kit (Uscn life Science, Wuhan, China). Sensitivities of assays were 0.069 ng/mL, 0.52 μg/mL, 2.9 pg/mL and 2.6 pg/mL for SIgA, IgG, IFN-γ and IL-4, respectively. For each immunity index, intra and inter assay coeffients of variation were <10% and <12%, respectively.

### Flow cytometric analysis of blood samples

Antibodies against the markers anti-mouse CD8a-PECy7, anti-mouse CD4-FITC and anti-mouse CD25-PE with corresponding isotype-matched controls were purchased from eBioscience (San Diego, CA, USA). For CD4^+^ T cells and CD8^+^ T cells, red blood cells were eliminated by incubation for 10 min with lysing buffer and then were centrifuged at 250 g for 5 min. In each flow cytometry tube, 200 μL blood was incubated with 10 μL CD4-FITC and 10 μL CD8a-PE-Cy7 for 20 min at 4 °C in the dark. Cell pellet were resuspended and washed with 2 mL phosphate buffer saline (PBS) by 250 g for 5 min. Washed cell pellets were then resuspended in 200 μL flow cytometry staining buffer for analysis. For CD4^+^CD25^+^ cells, 200 μL blood was incubated with 0.5 μL CD4-FITC and 0.6 μL CD25-PE for 20 min at 4 °C in the dark. After that, the samples were fixed and analyzed by flow cytometry.

### Statistical analyses

Statistical analyses were performed using SPSS 15.0 software. Data were expressed as mean ± SEM. One-way ANOVA followed by the correction of p values with Dunnett’s *post hoc test* was used to determine the significance of data. Longitudinal data were assessed using two-way ANOVA, where the between subjects factor was different doses of ceftriaxone and the within subjects factor was time point. P values < 0.05 was considered to be statistically significant.

## Additional Information

**How to cite this article:** Guo, Y. *et al*. Long-term use of ceftriaxone sodium induced changes in gut microbiota and immune system. *Sci. Rep.* **7**, 43035; doi: 10.1038/srep43035 (2017).

**Publisher's note:** Springer Nature remains neutral with regard to jurisdictional claims in published maps and institutional affiliations.

## Figures and Tables

**Table 1:** Sequence identities of PCR amplicons derived from DGGE gels.

Selected bands	Organism with highest sequence homology, sequence accession no. (% homology)	Bacterial Phyla	GeneBank accession number
1	Vagococcus entomophilus (99)	Enterococcus	NR_133886.1
2	Enterococcus hirae (99)	Enterococcus	NR_114783.2
3	Enterococcus pseudoavium (99)	Enterococcus	NR_114785.2
4	Enterococcus faecium (99)	Enterococcus	NR_102790.1
5	Clotridium thermopalmarium (99)	Clostridium	EF_639852.1
6	Enterococcus pseudoavium (99)	Enterococcus	NR_114785.2
7	Enterococcus faecium (99)	Enterococcus	NR_102790.1
8	Robinsoniella peoriensis (100)	Robinsoniella	NR_041882.1
9	Anaeroplasma bactoclasticum (94)	Anaeroplasma	NR_044675.2
10	Anaeroplasma bactoclasticum (97)	Anaeroplasma	NR_029167.1
11	Enterococcus faecium (99)	Enterococcus	NR_102790.1
12	Robinsoniella peoriensis (99)	Robinsoniella	NR_041882.1
13	Escherichia fergusonii (99)	Escherichia	NR_074902.1
14	Anaeroplasma bactoclasticum (94)	Anaeroplasma	NR_029167.1
15	Anaeroplasma varium (94)	Anaeroplasma	NR_044663.2
16	Enterococcus faecium (99)	Enterococcus	NR_102790.1
17	Robinsoniella peoriensis (100)	Robinsoniella	NR_041882.1
18	Anaeroplasma bactoclasticum (94)	Anaeroplasma	NR_029167.1
19	Anaeroplasma varium (94)	Anaeroplasma	NR_044663.2
20	Enterococcus faecium (99)	Enterococcus	NR_102790.1
21	Robinsoniella peoriensis (100)	Robinsoniella	NR_041882.1
22	Bergeriella denitrificans (93)	Bergeriella	NR_114040.1
23	Anaeroplasma bactoclasticum (93)	Anaeroplasma	NR_029167.1
24	Anaeroplasma varium (94)	Anaeroplasma	NR_044663.2
25	Enterococcus faecium (99)	Enterococcus	NR_102790.1
26	Robinsoniella peoriensis (100)	Robinsoniella	NR_041882.1
27	Escherichia fergusonii (100)	Escherichia	NR_074902.1

## References

[b1] Dethlefsen L., Huse S., Sogin M. L. & Relman D. A. The pervasive effects of an antibiotic on the human gut microbiota, as revealed by deep 16S rRNA sequencing. PLoS biology 6, e280, doi: 10.1371/journal.pbio.0060280 (2008). 19018661 PMC2586385

[b2] Dethlefsen L. & Relman D. A. Incomplete recovery and individualized responses of the human distal gut microbiota to repeated antibiotic perturbation. Proceedings of the National Academy of Sciences of the United States of America 108 Suppl 1, 4554–4561, doi: 10.1073/pnas.1000087107 (2011). 20847294 PMC3063582

[b3] Antonopoulos D. A. . Reproducible community dynamics of the gastrointestinal microbiota following antibiotic perturbation. Infection and immunity 77, 2367–2375, doi: 10.1128/IAI.01520-08 (2009). 19307217 PMC2687343

[b4] Buffie C. G. . Profound alterations of intestinal microbiota following a single dose of clindamycin results in sustained susceptibility to Clostridium difficile-induced colitis. Infection and immunity 80, 62–73, doi: 10.1128/IAI.05496-11 (2012). 22006564 PMC3255689

[b5] Ubeda C. . Vancomycin-resistant Enterococcus domination of intestinal microbiota is enabled by antibiotic treatment in mice and precedes bloodstream invasion in humans. The Journal of clinical investigation 120, 4332–4341, doi: 10.1172/JCI43918 (2010). 21099116 PMC2993598

[b6] Hill D. A. & Artis D. Intestinal bacteria and the regulation of immune cell homeostasis. Annual review of immunology 28, 623–667, doi: 10.1146/annurev-immunol-030409-101330 (2010). PMC5610356 20192812

[b7] Ohwaki M., Yasutake N., Yasui H. & Ogura R. A comparative study on the humoral immune responses in germ-free and conventional mice. Immunology 32, 43–48 (1977). 321340 PMC1445201

[b8] Zeng M. Y. . Gut Microbiota-Induced Immunoglobulin G Controls Systemic Infection by Symbiotic Bacteria and Pathogens. Immunity 44, 647–658, doi: 10.1016/j.immuni.2016.02.006 (2016). 26944199 PMC4794373

[b9] Cebra J. J., Periwal S. B., Lee G., Lee F. & Shroff K. E. Development and maintenance of the gut-associated lymphoid tissue (GALT): the roles of enteric bacteria and viruses. Developmental immunology 6, 13–18 (1998). 9716901 10.1155/1998/68382 PMC2276005

[b10] Larsson E. . Analysis of gut microbial regulation of host gene expression along the length of the gut and regulation of gut microbial ecology through MyD88. Gut 61, 1124–1131, doi: 10.1136/gutjnl-2011-301104 (2012). 22115825 PMC3388726

[b11] Lathrop S. K. . Peripheral education of the immune system by colonic commensal microbiota. Nature 478, 250–254, doi: 10.1038/nature10434 (2011). 21937990 PMC3192908

[b12] Bosi P. . Feed supplemented with 3 different antibiotics improved food intake and decreased the activation of the humoral immune response in healthy weaned pigs but had differing effects on intestinal microbiota. Journal of animal science 89, 4043–4053, doi: 10.2527/jas.2010-3311 (2011). 21724943

[b13] Hill D. A. . Commensal bacteria-derived signals regulate basophil hematopoiesis and allergic inflammation. Nature medicine 18, 538–546, doi: 10.1038/nm.2657 (2012). PMC3321082 22447074

[b14] Li M. . Fecal microbiota transplantation and bacterial consortium transplantation have comparable effects on the re-establishment of mucosal barrier function in mice with intestinal dysbiosis. Frontiers in microbiology 6, 692, doi: 10.3389/fmicb.2015.00692 (2015). 26217323 PMC4493656

[b15] Kim Y. . Effects of dexmedetomidine on the ratio of T helper 1 to T helper 2 cytokines in patients undergoing laparoscopic cholecystectomy. Journal of clinical anesthesia 26, 281–285, doi: 10.1016/j.jclinane.2013.11.018 (2014). 24856796

[b16] Zhang Y. . NF-kappaB-dependent cytokines in saliva and serum from patients with oral lichen planus: a study in an ethnic Chinese population. Cytokine 41, 144–149, doi: 10.1016/j.cyto.2007.11.004 (2008). 18222093

[b17] Matsuoka K. & Kanai T. The gut microbiota and inflammatory bowel disease. Seminars in immunopathology 37, 47–55, doi: 10.1007/s00281-014-0454-4 (2015). 25420450 PMC4281375

[b18] Carroll I. M., Ringel-Kulka T., Siddle J. P. & Ringel Y. Alterations in composition and diversity of the intestinal microbiota in patients with diarrhea-predominant irritable bowel syndrome. Neurogastroenterology and motility: the official journal of the European Gastrointestinal Motility Society 24, 521–530, e248, doi: 10.1111/j.1365-2982.2012.01891.x (2012). 22339879 PMC3975596

[b19] Ahn J. . Human gut microbiome and risk for colorectal cancer. Journal of the National Cancer Institute 105, 1907–1911, doi: 10.1093/jnci/djt300 (2013). 24316595 PMC3866154

[b20] Kang D. W. . Reduced incidence of Prevotella and other fermenters in intestinal microflora of autistic children. PloS one 8, e68322, doi: 10.1371/journal.pone.0068322 (2013). 23844187 PMC3700858

[b21] Mosca A., Leclerc M. & Hugot J. P. Gut Microbiota Diversity and Human Diseases: Should We Reintroduce Key Predators in Our Ecosystem? Frontiers in microbiology 7, 455, doi: 10.3389/fmicb.2016.00455 (2016). 27065999 PMC4815357

[b22] Lakticova V., Hutton-Thomas R., Meyer M., Gurkan E. & Rice L. B. Antibiotic-induced enterococcal expansion in the mouse intestine occurs throughout the small bowel and correlates poorly with suppression of competing flora. Antimicrobial agents and chemotherapy 50, 3117–3123, doi: 10.1128/AAC.00125-06 (2006). 16940110 PMC1563521

[b23] van Hal S. J. . Evolutionary dynamics of Enterococcus faecium reveals complex genomic relationships between isolates with independent emergence of vancomycin resistance. Microbial genomics 2, doi: 10.1099/mgen.0.000048 (2016). PMC5049587 27713836

[b24] National Nosocomial Infections Surveillance, S. National Nosocomial Infections Surveillance (NNIS) System Report, data summary from January 1992 through June 2004, issued October 2004. American journal of infection control 32, 470–485, doi: 10.1016/S0196655304005425 (2004). 15573054

[b25] Arias C. A. & Murray B. E. The rise of the Enterococcus: beyond vancomycin resistance. Nature reviews. Microbiology 10, 266–278, doi: 10.1038/nrmicro2761 (2012). 22421879 PMC3621121

[b26] Ferraris L., Aires J. & Butel M. J. Isolation of Robinsoniella peoriensis from the feces of premature neonates. Anaerobe 18, 172–173, doi: 10.1016/j.anaerobe.2011.11.007 (2012). 22155447

[b27] Jiang S. . Effects of Marsdenia tenacissima polysaccharide on the immune regulation and tumor growth in H22 tumor-bearing mice. Carbohydrate polymers 137, 52–58, doi: 10.1016/j.carbpol.2015.10.056 (2016). 26686104

[b28] Levings M. K. . Human CD25^+^CD4^+^ T suppressor cell clones produce transforming growth factor beta, but not interleukin 10, and are distinct from type 1 T regulatory cells. The Journal of experimental medicine 196, 1335–1346 (2002). 12438424 10.1084/jem.20021139 PMC2193983

[b29] Sakaguchi S., Sakaguchi N., Asano M., Itoh M. & Toda M. Pillars article: immunologic self-tolerance maintained by activated T cells expressing IL-2 receptor alpha-chains (CD25). Breakdown of a single mechanism of self-tolerance causes various autoimmune diseases. J. Immunol. 1995. Journal of immunology 186, 3808–3821 (2011). 21422251

[b30] Strzepa A. . Oral treatment with enrofloxacin early in life promotes Th2-mediated immune response in mice. Pharmacological reports: PR 68, 44–50, doi: 10.1016/j.pharep.2015.07.002 (2016). 26721350

[b31] Yeom M. . Oral administration of Lactobacillus casei variety rhamnosus partially alleviates TMA-induced atopic dermatitis in mice through improving intestinal microbiota. Journal of applied microbiology 119, 560–570, doi: 10.1111/jam.12844 (2015). 25968453

[b32] Atarashi K. . Induction of colonic regulatory T cells by indigenous Clostridium species. Science 331, 337–341, doi: 10.1126/science.1198469 (2011). 21205640 PMC3969237

[b33] Round J. L. & Mazmanian S. K. Inducible Foxp3+ regulatory T-cell development by a commensal bacterium of the intestinal microbiota. Proceedings of the National Academy of Sciences of the United States of America 107, 12204–12209, doi: 10.1073/pnas.0909122107 (2010). 20566854 PMC2901479

[b34] Joossens M. . Dysbiosis of the faecal microbiota in patients with Crohn’s disease and their unaffected relatives. Gut 60, 631–637, doi: 10.1136/gut.2010.223263 (2011). 21209126

